# UNMET NEED AMONG A NATIONAL SAMPLE OF OLDER ADULT HCBS USERS

**DOI:** 10.1093/geroni/igad104.1497

**Published:** 2023-12-21

**Authors:** Tetyana Shippee, Taylor Bucy, John Mulcahy, Eric Jutkowitz

**Affiliations:** University of Minnesota, Minneapolis, Minnesota, United States; University of Minnesota School of Public Health, Minneapolis, Minnesota, United States; University of Minnesota, Minneapolis, Minnesota, United States; Brown University, Providence, Rhode Island, United States

## Abstract

Long-term services and supports delivered in the home or community (home and community-based services) continue to be favored by consumers and policymakers alike. Given that HCBS is largely state-directed, there is considerable heterogeneity in program design and eligibility, and no standardized tool by which quality is measured. In the absence of the widespread use of standardized and uniform measures of HCBS quality, unmet need is poorly understood. We examined the relationship between services received and desired as a means of predicting unmet need among a national sample of older adult (65+) consumers of publicly funded HCBS (e.g., Medicaid, PACE, OAA) using data from the National Core Indicators - Aging and Disability survey. Our sample included 13,459 older community-dwelling adults, of which 60.0% were White, 23.2% were Black, and 17.0% of another race/ethnicity. Roughly 15% of older adults reported having AD/ADRD (54% White, 20% Black). Older adults with AD/ADRD were more likely to use personal care services (59.6%), followed by delivered meals (21.3%), and homemaker services (18.6%) compared to those without AD/ADRD. Adults with AD/ADRD desired more caregiver support services and transportation services vs what they were already receiving. Of 10% already receiving caregiver support services, approx. 9% desired additional support. Of 9.6% receiving transportation services, 7.3% desired additional support. We found variation in HCBS received and desired among older adults by both AD/ADRD status and race/ethnicity, suggesting gaps in unmet needs for these groups.

